# Health-related quality of life as measured with the Short-Form 36 (SF-36) questionnaire in patients with recurrent vulvovaginal candidiasis

**DOI:** 10.1186/s12955-016-0470-2

**Published:** 2016-04-29

**Authors:** Yu-Xia Zhu, Ting Li, Shang-Rong Fan, Xiao-Ping Liu, Yi-Heng Liang, Ping Liu

**Affiliations:** Department of Obstetrics and Gynecology, Peking University Shenzhen Hospital, Shenzhen, 518036 China; Shantou University Medical College, Shantou, Guangdong 515041 China; Shenzhen Key Laboratory of Gynecological Diagnostic Technology Research, Shenzhen, 518036 PR China; Department of Laboratory Science, Peking University Shenzhen Hospital, Shenzhen, PR China

**Keywords:** Recurrent vulvovaginal candidiasis, Health-related quality of life, SF-36, Candida

## Abstract

**Background:**

Recurrent vulvovaginal candidiasis (RVVC) has a poor therapeutic outcome and a severe impact on women and their partners, both physically and psychologically. Health-related quality of life (HRQOL) is significantly affected in patients with RVVC; however, little is known about HRQOL in patients with this disease. In this study, we aim to identify the clinical and mycological characteristics of women with RVVC and the effects of RVVC on women’s HRQOL.

**Methods:**

We designed this study as a comparative cross-sectional study. The Short-Form Health Survey (SF-36) was used to measure HRQOL in 102 patients with RVVC and 101 women seeking general health care (controls). RVVC was defined as four or more episodes of proven VVC in the previous 12-month period. VVC was defined as vulvar itching, burning, erythema, vaginal discharge, pseudohyphae or blastoconidia on a wet 10 % potassium hydroxide (KOH)-treated vaginal slide and a positive Candida culture. Group comparisons were conducted with independent samples *t* test. Correlation analysis was performed on the variables.

**Results:**

The mean age at first diagnosis of the patients with RVVC was 30.96 years (SD 5.38), and the mean age of the controls was 29.75 years (SD 5.83; *p* > 0.05). The duration of the patients’ complaints varied from 6 months to 10 years, with a mean duration of 22.28 (±21.75) months. The most common complaints were increased vaginal discharge (102 cases, 100 %), itching (97 cases, 95.1 %), dyspareunia (65 cases, 63.7 %), burning (79 cases, 77.5 %) and erythema (25 cases, 24.5 %). *C. albicans* was the predominant Candida species (86 strains, 84.3 %) in the patients, followed by *C. glabrata* (12 strains, 11.8 %). *C. parapsilosis* (1 strain, 0.9 %), *C. tropicalis* (1 strain, 0.9 %), *C. krusei* (1 strain, 0.9 %) and *C. lusitaniae* (1 strain, 0.9 %). The mean SF-36 dimension scores for physical function, role physical, bodily pain, general health, vitality, social functioning, role emotional, and mental health were significantly lower in the patients with RVVC than in the controls (85.20, 61.39, 77.79, 54.95, 53.17, 67.89, 52.48 and 59.17 vs. 90.20, 80.87, 87.08, 67.38, 59.69, 79.86, 68.01 and 65.38). The physical composite and mental composite scores of the patients with RVVC were 63.06 and 64.87, respectively, which were lower than those of the controls (75.01 and 74.87; *p* < 0.05).

**Conclusions:**

Nearly all of the patients with RVVC had clinical symptoms. In our sample, RVVC was mainly caused by *C. albicans*. RVVC has negative effects on women’s HRQOL, as indicated by lower physical and mental composite scores among the RVVC group compared with controls.

## Background

Recurrent vulvovaginal candidiasis (RVVC) defined as four or more episodes of vulvovaginal candidiasis (VVC) in a 12-month period and is estimated to occur in up to 5–8 % of women of child-bearing ages [1–3]. RVVC have a poor therapy outcome and have a severe impact on women and their partners, both physically and psychologically [4–11]. The main symptoms of RVVC, which was mainly caused by *C. albicans*, are itching, an abnormal vaginal discharge and painful sexual intercourse [6, 10, 12]. The symptoms and therapy of RVVC seriously interfered with sexual and emotional relationships of the affected women [13, 14]. Health-related quality of life (HRQoL) is a study area that has attracted increasing interests over the past two decades. Short-form 36 questionnaire (SF-36), which is an instrument for assessing quality of life, has been used in study to assess the quality of life of general population and the population with special chronic diseases world-wide [15–25]. We use SF-36 questionnaire to investigate the impact of RVVC on the quality of life of the affected women [26–28].

## Methods

### Participants

A total of 102 consecutive patients with RVVC who attended the RTI clinic of Peking University Shenzhen Hospital from 2014 to 2015 were invited to participate in this study by completing data forms after their appointments. The participants who came for general health care that is not concerned about other diseases, which included as the following exclusions criteria, comprised the control group (*n* = 101). The inclusion criteria of patients were: aged 18–50 years-old, generally healthy women with RVVC. Patients were excluded from entry if they: (1) had any other sexually transmitted disease or gynaecological abnormality requiring treatment; (2) had serious health problems known to effect quality of life such as diabetes mellitus. The comparison group within the same age range was recruited from the same clinic, where they attended for health care, in order to represent women from a general population, not concerned about reproductive tract infection (RTI) or other diseases. RTI including bacterial vaginosis, trichomoniasis and vulvovaginal candidiasis, were examed for all patients with RVVC and controls. The study was approved by Peking University Shenzhen Hospital Medical Ethics Committee and all participants provided written informed consent prior to study.

We administered the questionnaires while the women were waiting for their laboratory test reports. All of the patients were seen in the clinic by one of the authors (YXZ or TL), who made the final RVVC diagnosis and prescribed treatment. Nearly all the patients with RVVC and the controls completed the SF-36 questionnaire on their own at one separate room by thinking back to their most recent VVC episodes. Only several participants completed the questionnaire under the help of the authors (YXZ or TL). Additional information about demographic and clinical characteristics, Candida species, and other information pertaining to the RVVC was also collected by use of a study form.

### Case definition and Candida identification

VVC was defined as the presence of vulvar itching, vaginal discharge, blastoconidia and pseudohyphae on a wet 10 % KOH-treated vaginal slide and a positive Candida culture. The severity of each symptom such as itching, burning, erythema, and discharge was assigned a score on the following scale: 0 = absent; 1 = mild; 2 = moderate; 3 = severe. Patients with a severity score of 7 or greater were designated as severe vulvovaginal candidiasis. RVVC was defined as four or more episodes of proven infection in the previous 12-month period. Specimens were plated on CHROMagar (Biocell Laboratory Ltd, Zhengzhou, China) for 24 to 48 h at 37 °C in ambient air. Strains were identified using the standardized API Candida system (bioMerieux, Marcy l’Etoile, France).

### SF-36 questionnaire

The SF-36 questionnaire consists of 36 items, which are used to calculate eight subscales: physical functioning (PF), role physical (RP), bodily pain (BP), general health (GH), vitality (VT), social functioning (SF), role emotional (RE), and mental health (MH). The first four scores can be summed to create the physical composite score (PCS), while the last four can be summed to create the mental composite score (MCS). Scores for the SF-36 scales range between 0 and 100, with higher scores indicating a better HRQOL [25–27].

### Statistical analysis

Statistical analysis was performed using Statistical Package for the Social Sciences 21.0 (SPSS Inc., Chicago, IL, USA). Descriptive statistics were used to summarize the patients’ baseline characteristics. The data are presented as number and percentage unless otherwise indicated. Group comparisons were conducted with independent samples *t* test. Pearson’s correlation was performed to test the correlation among continuous variables. Spearman’s correlation was performed to test the correlation among rank variables. All statistical tests were two-sided (α = 0.05). *P* < 0.05 was accepted as significant.

## Results

### The characteristics of the patients with RVVC and controls

The 102 patients with RVVC and 101 controls who fulfilled the inclusion criteria were admitted to the study. The mean age at first diagnosis of the patients with RVVC and the controls was 30.96 (SD 5.38) and 29.75 (SD 5.83) years, respectively. The duration of the patients’ complaints varied from 6 months to 10 years, with a mean duration of 22.28 (SD 21.75) months. The most common complaints were increased vaginal discharge (102 cases, 100 %), itching (97 cases, 95.1 %), dyspareunia (65 cases, 63.7 %), burning (79 cases, 77.5 %) and erythema (25 cases, 24.5 %). Of the 102 patients with RVVC who were recruited to participate, one patient had a history of drug allergies, one patient had diabetes mellitus complications, and one patient reported a male partner with symptoms of a genital Candida infection. No patients were infected with HIV. One hundred two Candida strains were recovered from the vaginal swabs of the patients with RVVC. *C. albicans* was the predominant Candida species (86 strains, 84.3 %), followed by *C. glabrata* (12 strains, 11.8 %), *C. parapsilosis* (1 strain, 0.9 %), *C. tropicalis* (1 strain, 0.9 %), *C. krusei* (1 strain, 0.9 %) and *C. lusitaniae* (1 strain, 0.9 %).

### Health-related quality of life in patients with RVVC

The mean SF-36 dimension scores for physical function, role physical, bodily pain, general health, vitality, social functioning, role emotional, and mental health were significantly lower in the patients with RVVC than in the controls (85.20, 61.39, 77.79, 54.95, 53.17, 67.89, 52.48 and 59.17 vs. 90.20, 80.87, 87.08, 67.38, 59.69, 79.86, 68.01 and 65.38), *p* < 0.05. The PCS and MCS of the patients with RVVC were 63.06 and 64.87, which were lower than those of the controls (75.01 and 74.87), *p* < 0.05, Table 1.

**Table 1:** Findings of SF 36 quality of life scale in patients with RVVC and controls^a^

SF-36 scales	RVVC group (*n* = 102)		Control group (*n* = 101)			
	Mean	SD	Mean	SD	t	*P* value
PF	85.20	16.99	90.20	12.12	2.401	0.017
RP	61.39	36.66	80.87	31.25	4.038	0.000
BP	77.79	18.33	87.08	15.01	3.946	0.000
GH	54.95	18.66	67.38	16.48	5.026	0.000
VT	53.17	14.89	59.69	14.58	3.121	0.002
SF	67.89	17.89	79.86	15.92	5.030	0.000
RE	52.48	40.37	68.01	40.10	2.730	0.007
MH	59.17	15.74	65.38	15.57	2.801	0.006
PCS	63.05	20.28	75.01	18.78	4.382	0.000
MCS	64.87	13.92	74.87	12.22	5.378	0.000

^a^Independent samples *t* test. *RVVC* recurrent vulvovaginal candidiasis

The PCS and MCS scores of the patients with RVVC have no correlations with Candida species and VVC score, which were based on the severity of each symptom including itching, burning, erythema, and discharge, Tables 2 and 3. The SF 36 quality of life scale PCS and MCS score were lower in patients with RVVC caused by *C. albicans* and with high VVC score, *p* > 0.05.

**Table 2:** Bivariate correlations between SF 36 quality of life scale PCS and MCS with characteristics of patients with RVVC

SF 36 scale and characteristics of RVVC	1	2	3	4	5	6	7	8
1. PCS#	1							
2. MCS#	0.736**	1						
3. Ages of patients#	0.045	0.075	1					
4. VVC score#	−0.056	0.010	0.089	1				
5. Itching §	0.028	0.017	0.183	0.507**	1			
6. Burning §	0.087	0.086	0.134	0.510**	0.026	1		
7. Erythema §	−0.035	0.002	0.052	0.532**	−0.013	0.192	1	
8. Vaginal discharge §	−0.076	0.012	−0.174	0.767**	0.090	0.218*	0.386**	1

#Pearson’s correlation was performed to test the correlation among continuous variables. § Spearman’s correlations was performed to test the correlation among rank variables. Significance correlation at *p < 0.05 level and **p < 0.01 level (2-tailed). *MCS* Mental composite score, *PCS* Physical composite score, *RVVC* recurrent vulvovaginal candidiasis

**Table 3:** Comparison of SF 36 quality of life scale PCS and MCS with VVC score and Candida species in patients RVVC^a^

SF-36 scales	VVC score		Candida species	
	~6	>6	*C. albicans*	*Non albicans*
*n*	87	15	86	16
PCS, Mean (SD)	63.4254 (20.49504)	58.2576 (20.03453)	64.5119 (14.02028)	66.6544 (13.74386)
t	0.789		0.474	
*P* value	0.432		0.639	
MCS, Mean (SD)	65.1078 (13.59153)	62.3636 (17.07332)	62.4548 (21.12199)	64.5588 (15.60170)
t	0.613		0.576	
*P* value	0.541		0.566	

^a^Independent Samples *t* test. *MCS* Mental composite score, *PCS* Physical composite score, *RVVC* recurrent vulvovaginal candidiasis

**Table 4:** Findings of SF 36 quality of life scale in patients and controls

Population		PCS	MCS	References
Adults of the Chinese general population	Normal weight	77.5	73.4	Zhu, 2015 [25]
	Underweight	75.4	71.8	
	Overweight	78.0	73.9	
	Class I obese	78.4	75.1	
	Class II obese	76.8	75.0	
Overweight older adults, Stockholm County	Swedish physical activity intervention baseline	80	84	Olsson, 2015 [22]
	Swedish physical activity controls baseline	83	89	
Female rural-to-urban migrants, Chinese female		76.79	68.59	Wang, 2015 [24]
Patients with benign breast lumps, Chinese		75.42	68.70	Lou, 2015 [20]
Knee osteoarthritis patient, Chinese		70.37	80.94	Pang, 2015 [23]
Type 2 diabetes, Chinese		67.4	64.9	Hu, 2015 [16]
Caregiver spouses of veterans with bilateral lower extremity amputations	Study group	70	64	Moradi, 2015 [21]
	Controls: general population	73	66	
Patients at Risk for CVD in Uruguay, female		64.4	64.8	Clennin, 2015 [15]
College teachers, female		71.35	65.63	Liu, 2015 [18]
Chinese psychiatrists		79.78	71.50	Liu, 2015 [19]
Pre-diabetes patients,	Normal weight	88.01	85.52	Ibrahim, 2014 [17]
	Overweight	86.78	85.55	
	Obese	78.59	83.06	
	Total	81.83	83.85	
Patients with RVVC and controls	Controls: general population	75.01	74.87	This study
	RVVC	63.05	64.87	

*CVD* cardiovascular disease, *PCS* Physical component summary, *MCS* Mental component summary

## Discussion

### Characteristics of the women with RVVC

The main symptoms of yeast infections are inflammation, itching, abnormal vaginal discharge and painful sexual intercourse and urination. Such symptoms cause variable but often severe discomfort and pain. Most of the patients with RVVC have no precipitating factor. All of our patients with RVVC had clinical symptoms. The mean duration of RVVC in our patients was 22.28 months. *C. albicans* was the predominant Candida species (86 strains, 84.3 %), followed by *C. glabrata* (12 strains, 11.8 %); these findings are consistent with previous reports [6, 10, 12].

### Health-related quality of life

The clinical impression is that patients with RVVC, despite current treatment options, suffer from a substantially impaired HRQOL, but quantifiable evidence to support this impression is scarce. Nyirjesy et al. [14] applied several validated pain, stress and depression measurements to a population of physician-diagnosed patients (*N* = 38) and found that 29 % had clinical depression. Aballéa et al. [8] studied the health status and HRQOL of women with RVVC in Europe and the USA. A total of 620 women with RVVC were selected to complete the EQ-5D questionnaire and the SF-36 questionnaire. Of these women, 68 % reported depression/anxiety problems during acute episodes and 54 % reported depression/anxiety when they were not experiencing episodes, compared with less than 20 % for the general population. All SF-36 domain scores were significantly below the general population norms, but the mental health domains were the most affected. The results indicate that HRQOL is substantially diminished both during acute episodes and outside of these episodes in women with RVVC.

The SF-36 is commonly used as a generic measure to assess HRQOL. It has gained popularity as a means of evaluating outcomes in a wide variety of patient groups and surveys [15–25]. The findings of the SF-36 quality of life scale in patients and controls from previous study were shown on Table 4. Our current study results showed that PCS and MCS of patients with RVVC were 63.05 and 64.87, which were lower than those in controls (75.01 and 74.87). The PCS and MCS of control in our study are similar to those of normal controls from a Chinese study (PCS: 75.01 vs 77.5; MCS: 74.87 vs 73.4), however, are lower than those of normal controls from a Malaysia study (PCS: 75.01 vs 88.01; MCS: 74.87 vs 85.52) [17, 25]. The PCS of patients with RVVC in our study arelower than those of patients with type 2 diabetes (63.05 vs 67.4). The MCS of patients with RVVC in our study are similar to those of patients with type 2 diabetes (64.87 vs 64.9) [16]. The results of our study were consistent with findings by Aballéa et al., who also reported a stronger impact of RVVC on mental health than on physical health using SF-36, albeit to a somewhat lesser extent [8]. Our data support the impression that the subjective health status and HRQOL in women with RVVC are significantly worse than in the general population. An important question raised by the study (as well as previous studies) is whether the reduction in HRQOL among women with RVVC is attributable to RVVC or associated comorbidities. In our current study, the SF 36 quality of life scale PCS and MCS score were lower (but without significantly statistical difference) in patients with RVVC caused by *C. albicans*, which was more virulence, and with high VVC score [1]. This suggests the reduction in HRQOL among women with RVVC may be attributable to the comorbidities of the infection. The factor(s) which exactly affect the PCS and MCS scores of the patients with RVVC are unclear and should be further studied.

The limitations of this study were that for the SF-36 questions, we replaced “During the past 4 weeks …” with “Thinking back to your most recent VVC…” and the sample size was determined based on only previous studies.

## Conclusions

Nearly all of the patients with RVVC had clinical symptoms, and most had no precipitating factor. RVVC was mainly caused by *C. albicans*. RVVC had negative effects on the women’s HRQOL.

## Abbreviations

BP: bodily pain
CI: confidence interval
CVD: cardiovascular disease
GH: general health
HRQOL: health-related quality of life
MCS: mental component summary
MH: mental health
PCS: physical component summary
PF: physical functioning
RE: role emotional
RP: role physical
RVVC: recurrent vulvovaginal candidiasis
SF: social functioning
VT: vitality
